# The Tensile Strength and Damage Characteristic of Two Types of Concrete and Their Interface

**DOI:** 10.3390/ma13010016

**Published:** 2019-12-18

**Authors:** Qingchuan Shen, Wei Chen, Chao Liu, Wenjie Zou, Liufeng Pan

**Affiliations:** 1 School of Civil Engineering, Central South University, Changsha 410075, China; shenqc@csu.edu.cn (Q.S.); 174814341@csu.edu.cn (W.Z.); 2 National Engineering Laboratory of High-Speed Railway Construction Technology, Changsha 410004, China; 3 School of Civil Engineering, Guangzhou University, Guangzhou 510006, China; chaoliu@gzhu.edu.cn (C.L.); 2111816189@e.gzhu.edu.cn (L.P.)

**Keywords:** concrete, interface, fiber reinforced interface, tensile splitting test, finite element analysis

## Abstract

In this study, tensile splitting tests and corresponding numerical simulations are performed on high strength concrete, low strength concrete, the interface between the two types of concrete, and the interface reinforced by steel fiber, respectively. The tensile splitting strength, crack initiation load, and damage characteristics are analyzed based on experiment data and fracture surface of samples. It can be concluded that tensile splitting strength and crack initiation load have the descending order of ‘HT’ (high strength concrete sample) > ‘LT’ (low strength concrete sample) > ‘FT’ (interface reinforced by steel fiber) > ‘OT’ (interface). The tensile splitting strength is related not only to the roughness of the fracture surface, but also to the percentage of fractured aggregates. The steel fiber can increase initiation cracking load, peak load and residual strength of the interface. The existing of interface composited by two types of material can cause asymmetric distribution of the stress state, even if geometry and the load are symmetrical for samples.

## 1. Introduction

The tensile strength and tensile damage characteristics of concrete/steel fiber reinforced concrete have been studied for decades due to their great importance in structural design and safety analysis. Splitting tensile test methods such as ASTM C-496 [1], ISO 4108 [2], BS 1881-117 [3], etc., are frequently adopted to measure the tensile strength and investigate the corresponding fracture characteristics of concrete materials [4,5]. For common concrete, according to quasi-static tensile splitting tests, the influences of water-cement ratios, porous structure, types of cement, aggregates sizes and samples sizes on the tensile strength of concrete have been extensively researched [6,7,8,9]. Meanwhile, the dynamic tensile fracture pattern and mechanical response of concrete has been studied by the dynamic Brazilian test [10], which has shown that the impact velocity plays a significant role in the failure pattern of concrete specimens. Through building mechanical models, Carmona and Aguado [11] have indirectly determined the tensile stress–strain curve of concrete with a splitting tensile test, Hoang et al. [12] have investigated the crack propagation process in the splitting plane and obtained the distribution of residual tensile strength as crack propagation, and Olesen et al. [13] have analyzed splitting tensile fracture and the compressive crushing/sliding failure during the test, In addition to experiments, numerical methods are also one of the most important methods for studying concrete structures, e.g., Giuseppe Fortunato et al. [14] and Luciano Ombres et al. [15] used a nonlinear constitutive relation (concrete damage plastic model) in a numerical model to study the mechanical and damage behavior of concrete structures under load. For steel fiber reinforced concrete, Prisco et al. [16] have identified corresponding post-cracking behavior, Abrishambaf et al. [17] have investigated the tensile stress-crack width law during post-cracking stage, and Boulekbache et al. [18] have studied the post failure mechanism of fiber reinforced concrete during splitting test based on digital image correlation. In addition, Olivito and Zuccarello [19] have studied the tensile strength of steel fiber reinforced concrete with respect to fiber content and mix-design variations, and Denneman et al. [20] have obtained a close estimate of the true tensile strength of fiber reinforced concrete from an adjusted tensile splitting test procedure, Shalchy and Askarinejad et al. [21,22] have studied the nanostructure of the cement/fiber interfaces, and the corresponding mechanical properties.

In addition to the aforementioned studies, the tensile mechanical behavior of concrete-concrete interface is also a research focus for scientists. Generally, the interface, which is weaker than both sides of materials, widely exists in repaired structures [23,24], composite structures [25] and Chinese high-speed railway track slab structures [26]. Based on splitting tensile tests, Tschegg and Stanzl [27] have measured the adhesive power of interface between old-new concretes, and Tayeh et al. [28] have investigated characteristics of the interface between old concrete and steel fiber reinforced concrete. Chandra Kishen and Subba Rao [25] have analyzed the fracture properties of concrete-concrete, transversely cold jointed interface beams. Shah and Kishen [29,30] have studied the fracture behavior of concrete-concrete interface by acoustic emission technique and analyzed nonlinear fracture properties of the interface.

**Figure 1:**
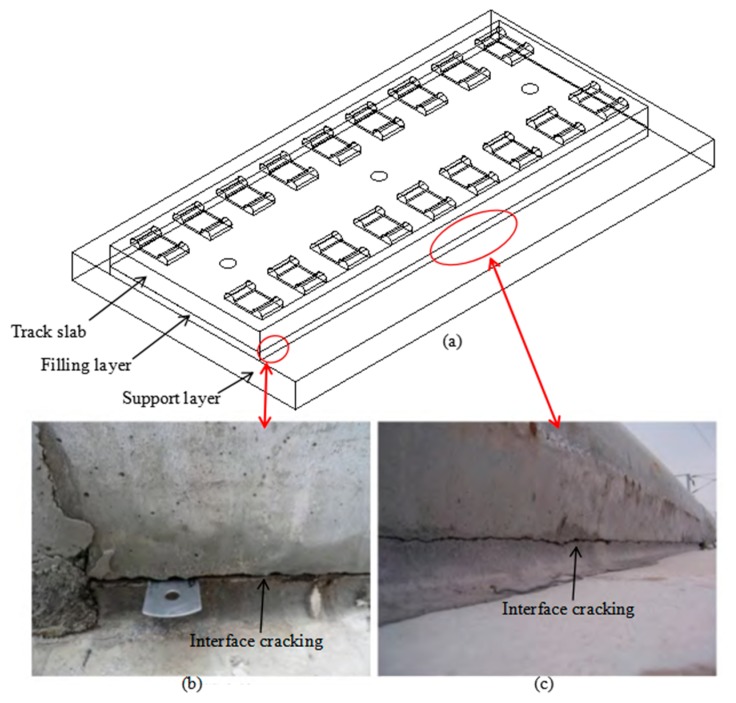
High-speed railway track slab structure and interface cracking, (**a**) schematic figure of the structure, (**b**) indicated interface cracking at the end of the structure [31], (**c**) indicated interface cracking in the middle of the structure [31].

Although various studies have been carried out in this area, the performance of the interface between different material properties in concrete was largely overlooked. First, the existing of interface between two types of concrete will cause a reduction of the strength; therefore, a quantitative comparison for the tensile strength value of interface and intact concretes is necessary and also a suitable numerical model to describe the mechanical response of the sample or structure with interface should be built. Second, the reinforcement method (such as using steel fiber) for the interface should be investigated. However, most of researchers [16,17,18,19,20,21,22] have considered to put steel fiber inside the concrete to form an intact sample or structure and ignored to study the reinforcement behavior of the steel fiber for the interface between different types of concretes (Similar to planting steel fiber in the interface). These respects are quite important for interface structure design and safety evaluation. In particular, some interface cracking phenomena have been observed in high-speed railway track slab structures during operations. As shown in Figure 1, the prefabricated track slab is installed on the support layer by pouring the filling layer on site. The filling layer is composed of asphalt mortar for CRTS I/II track slabs or concrete for CRTS III track slab (Figure 1a). Cracks generally emerged in the interface due to the reason that the interface is the weakest part in the whole structure (Figure 1b,c). Therefore, a systematic investigation is carried out in this study for quantitative analysis strength reduction of the interface and the reinforcement behavior of the steel fiber on the interface through a series of tensile splitting tests on four types of cubic concrete samples, including high strength concrete (used for track slab), low strength concrete (used for filling layer), cementation of low and high strength concretes (interface) and cementation of low and high strength concretes with steel fibers (interface reinforced by steel fiber which may be a possible improvement method).

The paper is organized as follows: first, samples preparation and experimental procedure are introduced; second, the tensile strength, initiation cracking point and damage properties of the concretes are analyzed and compared; third, the numerical simulations with finite element method (FEM) are conducted; finally, conclusions are drawn based on the tests and numerical simulations.

## 2. Experimental Work

### 2.1. Samples Preparation

**Figure 2:**
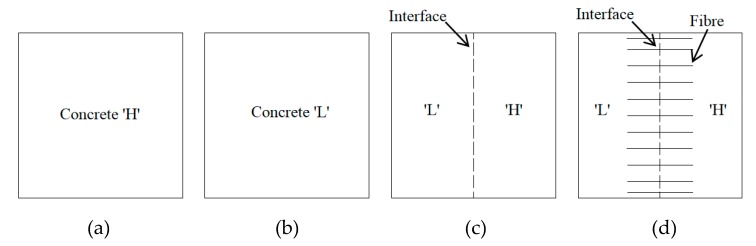
Cross sections of different samples, (**a**) sample fabricated by high strength concrete, (**b**) sample fabricated by low strength concrete, (**c**) sample with interface jointing two types of concrete, (**d**) sample with steel fiber reinforced interface jointing two types of concrete.

Cubical concrete samples are prepared with dimension of 150 mm × 150 mm × 150 mm. Cross sections for four types of samples are illustrated in Figure 2, where ‘H’ and ‘L’ stands for high strength and low strength concrete, respectively. The sample shown in Figure 2c,d is cemented by two types of concrete and thus an interface is formed. The interface in Figure 2d is reinforced by equally distributed steel fibers. The distance between two fibers is about 15 mm and 100 fibers are implanted passing through the interface. Mix proportions of concretes are listed in Table 1. The slag is the copper slag and the main contents includes Fe_2_O_3_, SiO_2_, Al_2_O_3_, and Cu_2_O. The apparent density and fineness modulus of the copper slag is 2650 kg/m^3^ and 3.3, respectively. The basic mechanical parameters for concrete ‘H’, concrete ‘L’ and steel fiber are listed in Table 2. The tests for basic mechanical parameters for concrete are according to ASTM C 39/C 39M-2005 [32], ASTM C 469-2002 [33] and ASTM C 138-2001 [34].

**Figure 3:**
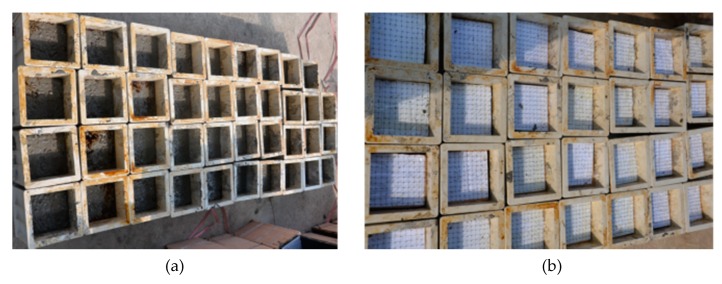

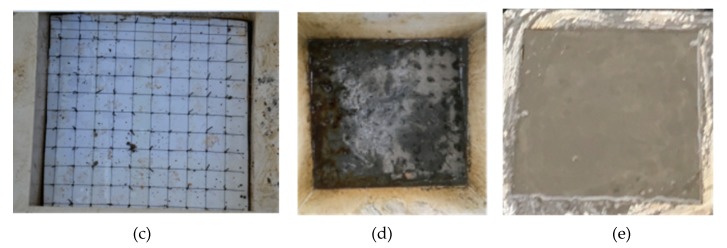
Manufacturing process for the samples with steel fiber reinforced interface, (**a**) filling half of cube with the high strength concrete, (**b**) implanting steel fiber, (**c**) magnified view of one of cubes, (**d**) removing the cardboard after initial-set of the high strength concrete, (**e**) filling the other part of the cube with the low strength concrete.

The manufacturing process for the samples with interface (Figure 2c) is as following. One half of cubic mold is filled with high strength concrete and after initial-set of the high strength concrete, the other half of cubic mold is filled with low strength concrete. The manufacturing process for the samples with steel fiber reinforced interface (Figure 2d) is shown in Figure 3. First, one half of cubic mold is filled with high strength concrete. Then, about 100 fibers are implanted into the concrete with the embedded depth of about 3 cm (The fibers are arranged in a cardboard with 15 mm × 15 mm grids in advance and then are pushed into concrete until the cardboard contacting the surface of concrete). After the initial-set of the high strength concrete, the cardboard is removed and the other half of cubic mold is filled with low strength concrete. Finally, all samples are demolded and are cured for 28 days.

### 2.2. Test Procedure

**Figure 4:**
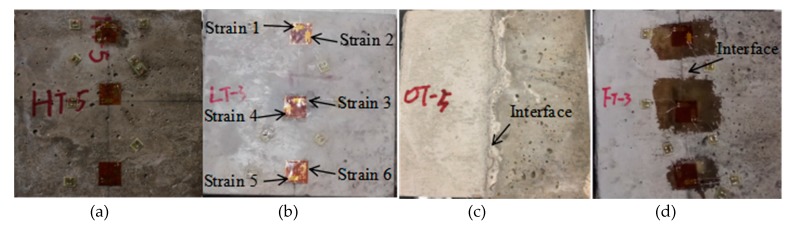
Different concrete samples, (**a**) high strength concrete cube, (**b**) low strength concrete cube, (**c**) interface without steel fiber, (**d**) interface reinforced by steel fiber.

**Figure 5:**
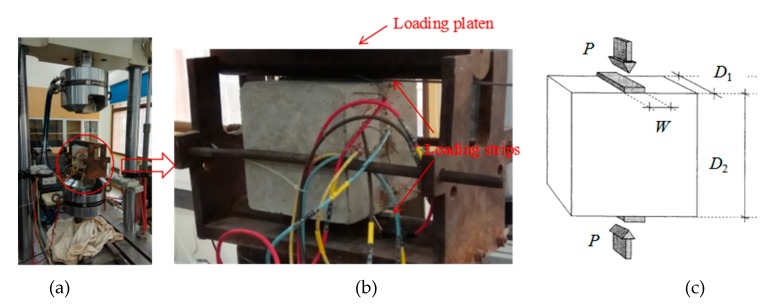
Illustration for tensile splitting tests, (**a**) MTS 322 testing system, (**b**) installed sample, (**c**) loading condition [4].

Before testing, the loading faces of samples are polished and strain gages are pasted on the samples. As shown in Figure 4, the strain gage 1, 3 and 5 measures the lateral strain and strain gage 2, 4 and 6 measures the vertical strain. All the tensile-splitting tests are conducted on an Mechanical Test and Simulation (MTS) 322 system (Figure 5). The load was transmitted through the loading strip (Figure 5c) with the width (W) of 6 mm [3]. The loading frame can sustain nominal axial load of 500 kN (error < 0.05%). The load, vertical displacement of loading platen, lateral strain and vertical strain of samples are recorded during tests. The testing procedure is in accordance with the standardized test method BS1881-117 with the loading rate of 1 kN/s [3].

## 3. Analysis of Experimental Results

### 3.1. Tensile Splitting Strength

The tensile splitting strength $\sigma_{ct}$ can be calculated by the following formula [3]:

(1) $$\sigma_{ct}=\frac{2P_{\max}}{\pi \times D_{1}\times D_{2}},$$

where $P_{\max}$ is the maximum vertical load; $D_{1}$ is the length of sample and $D_{2}$ is the cross-sectional dimension of the sample (shown in Figure 5c). For samples with the steel fibers, the area percentage of fiber $A_{f}$ on the interface can be calculated by the formula:

(2) $$A_{f}=\frac{n\times \pi \times r_{f}^{2}}{D_{1}\times D_{2}}=\frac{100\times \pi \times 0.5^{2}}{150\times 150}\approx 0.35\%,$$

where n is the number of fibers passing through interface and $r_{f}$ is the radius of fiber.

Test results for four types of samples are listed in Table 3, where ‘HT’,‘LT’,‘OT’ and ‘FT’ mean high strength concrete, low strength concrete, cementation of low and high strength concretes (interface) and cementation of low and high strength concretes with steel fibers (interface reinforced by steel fiber), respectively. According to Table 3, the tendency for tensile splitting strength can be characterized as ‘HT’ > ‘LT’ > ‘FT’ > ‘OT’. The average tensile splitting strength of ‘FT’ has increased about 10.9% compared with that of ‘OT’ (shown in Table 3) due to the reinforced effects of steel fiber. The ratio of average tensile splitting strength of ‘OT’ (interface) versus ‘HT’ and ‘OT’ versus ‘LT’ is 48.8% and 67.4%, respectively, verifying that the interface is weaker than both sides of materials.

### 3.2. Initial Cracking Point

**Figure 6:**
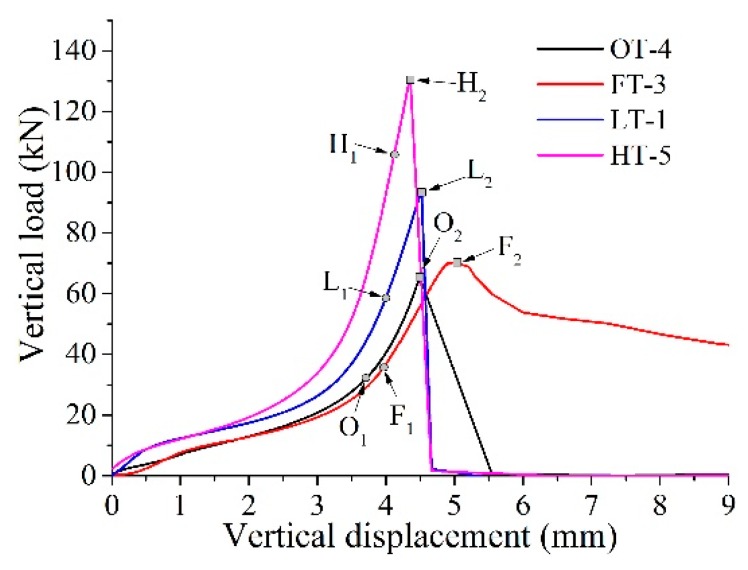
Vertical load versus vertical displacement.

The curves of vertical load versus vertical displacement for samples ‘HT-5’, ‘LT-1’, ‘FT-3’ and ‘OT-4’ are shown in Figure 6. ‘H_1_’, ‘L_1_’, ‘F_1_’ and ‘O_1_’ represents the initial cracking point for ‘HT-5’, ‘LT-1’, ‘FT-3’ and ‘OT-4’, respectively. ‘H_2_’, ‘L_2_’, ‘F_2_’ and ‘O_2_’ represent corresponding peak load points. The peak load points can be directly obtained from the testing system. However, the initial cracking points are chosen according to previous studies. For example, Carmona and Aguado [11] have concluded that the maximum tensile strain for concrete is between 0.00015 and 0.00025; Laranjeira [35] has indicated that the average maximum tensile strain is 0.0002. Therefore, the value of 0.0002 for lateral strain is considered to be the initial tensile cracking point for the samples. The values can be detected by analyzing the lateral strain data recorded in strain gages 1, 3 and 5 (Figure 4). Thus, the load corresponding to lateral strain 0.0002 can be located as initial cracking points in Figure 6. The value of initial cracking points for samples ‘HT-5’, ‘LT-1’, ‘FT-3’ and ‘OT-4’ are listed in Table 4. It can be concluded that the initial cracking points and peak loads has the tendency of ‘HT-5’ > ‘LT-1’ > ‘FT-3’ > ‘OT-4’, which means the interface has both the weakest strength and resistance to crack initiation. The test results also reveal the ability of steel fiber in reinforcing the interface to some extent.

**Figure 7:**
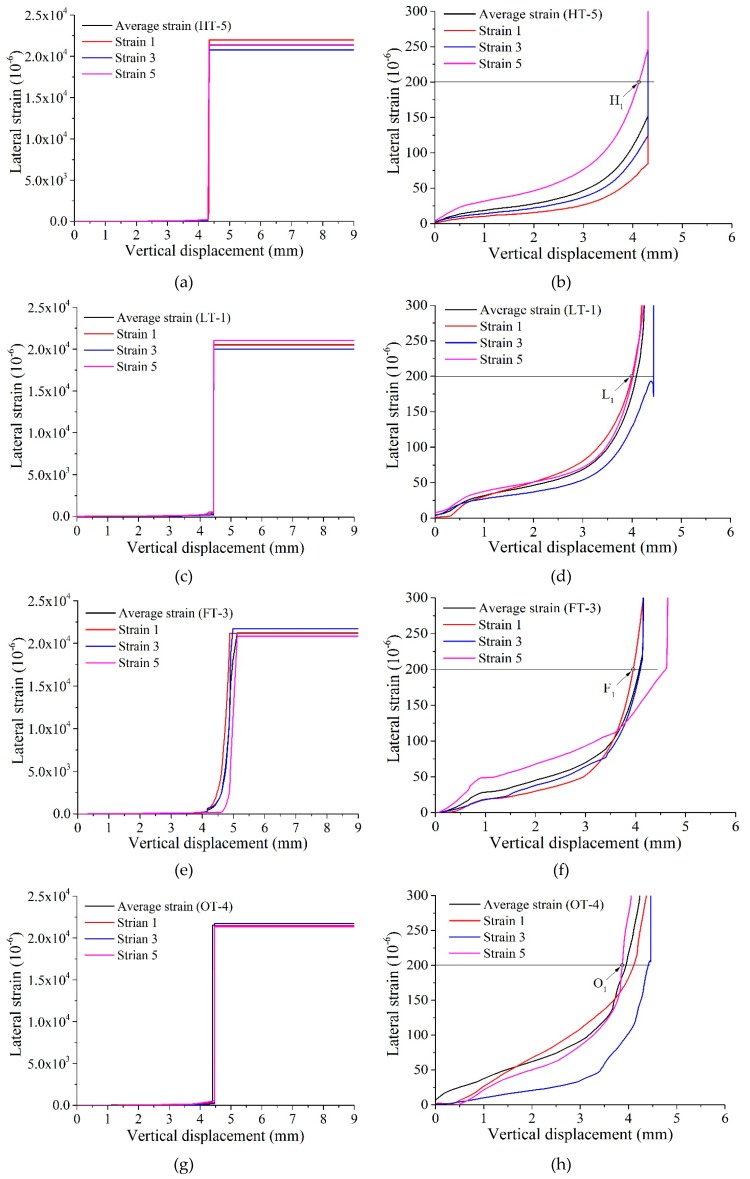
Lateral strain versus vertical displacement, (**a**), (**c**), (**e**) and (**g**) is the lateral strain versus vertical displacement for ‘HT-5’, ‘LT-1’, ‘FT-3’, ‘OT-4’, respectively. (**b**), (**d**), (**f**) and (**h**) are the corresponding enhanced view of lateral strain-vertical displacement curves for lateral strain smaller than 0.0003.

Lateral strain versus vertical displacement for samples ‘HT-5’, ‘LT-1’, ‘FT-3’ and ‘OT-4’ are illustrated in Figure 7. It is seen that the lateral strain firstly increases slowly with increasing vertical displacement. Once the loading process approach to peak point, the lateral strain increases sharply which indicates that cracks are emerging in the region of sample where strain gages are attached. Then the strain gages are damaged and lateral strain data cannot be updated and are kept at a big value (about 0.02). The initial cracking point for samples is also shown in Figure 7, where the value of lateral strain 0.0002 is chosen as maximum tensile strain for the samples. Obviously, before failure of strain gages, the lateral strain increases non-linearly with increasing vertical displacement (Figure 7b,d,f).

### 3.3. Damage Characteristics

**Figure 8:**
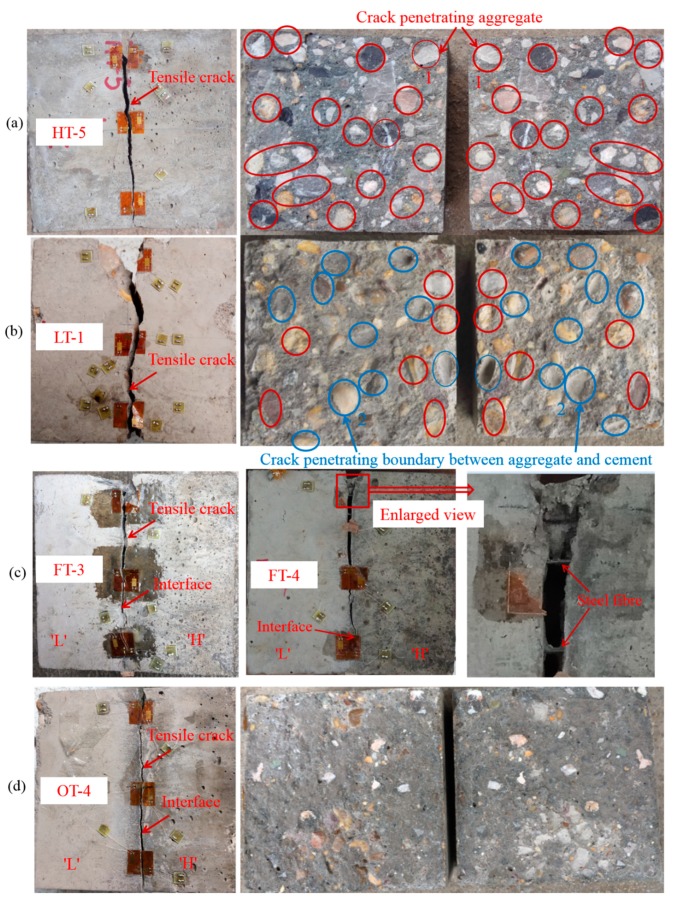
Fracture patterns of different samples, (**a**) sample ‘HT-5’, the red circles represent the cracked coarse aggregates, (**b**) sample ‘LT-1’, the blue circles represent the crack penetrates the boundary between the coarse aggregate and the cement (without damage to the coarse aggregate), (**c**) samples ‘FT-3’ and ‘FT-4’, (**d**) sample ‘OT-4’.

The different percentages of fractured areas are listed in Table 5. $A_{a}$,$A_{b}$, $A_{c}$ and $A_{total}$ is area of fractured surface of coarse aggregate, area of fractured boundary between coarse aggregate and cement, area of fractured cement and total area of cracking surface, respectively. The fracture patterns of different samples are illustrated in Figure 8. It is shown that typical tensile crack connects the loading points and passes through the samples which results in the final failure. However, the detailed fracture surfaces for different types of samples have different characteristics. For example, the crack can penetrate the aggregate which is indicated by the red circles in Figure 8a,b. In the middle and the right picture of Figure 8a, we mark two red circles with the number 1. It can be obviously observed in the number 1 circles that the coarse aggregate is damaged by the crack. As shown in the number 2 circles (Figure 8b), the left circle is the coarse aggregate and the right circle in the corresponding position is the cement. Therefore, it can be concluded the crack penetrates the boundary between the aggregate and the cement in this area and this type of damage is indicated by the blue circles. A plenty of coarse aggregates (about 30.9% in Table 5) are penetrated by the crack in sample ‘HT-5’ (Figure 8a). However, only a small part of coarse aggregates (about 12.0% in Table 5) are penetrated and crack occurs at the boundary between the rest aggregates and cement (about 15.5% in Table 5) in sample ‘LT-1’ (Figure 8b). For interface reinforced by steel fibers, a tensile crack along the interface emerges in samples ‘FT-3’ and ‘FT-4’, respectively. The difference of this sample to other types of sample is that two parts (high strength part and low strength part) of sample are still connected by the steel fibers. The width of crack in different position is indicated in Figure 8c. Difficulty has been encountered in attempting to manually separate them (the residual strength is high). In contrast, the fracture surface of ‘OT-4’ (interface without steel fiber) is smooth, where few aggregates (about 3.7% in Table 5) are damaged and the crack almost propagates through the cement (about 91% in Table 5). The damage information on cracking surfaces of different samples can explain the essential reason for the tendency of tensile splitting strength of ‘HT’ > ‘LT’ > ‘FT’ > ‘OT’. It can be concluded that the tensile splitting strength of concrete has close relation to both the roughness of cracking surface and fracturing percentage of aggregates. The roughness of cracking surface in sample ‘LT-1’ is bigger than that of sample ‘HT-5’, but the tensile splitting strength of sample ‘LT-1’ is smaller than that of sample ‘HT-5’ due to the influence of cracked aggregates (more aggregates are fractured in sample ‘HT-5’). The steel fiber can also increase the residual tensile strength due to the bridging effects (Figure 8c).

**Figure 9:**
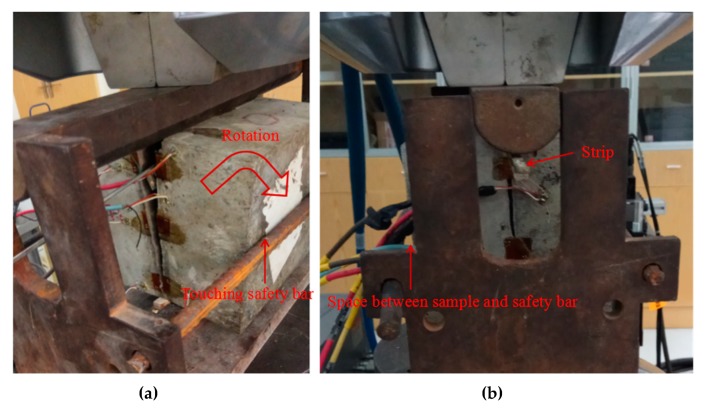
Post-failure state, (**a**) Sample without steel fiber, (**b**) Interface of sample reinforced by steel fiber.

The post-failure stage for samples ‘HT-5’, ‘LT-1’ and ‘OT-4’ are similar, which is featured by rapidly decrease of load with increasing vertical displacement (Figure 6). Actually, it is observed that this phenomenon is related to the occurrence of unbalance state of samples (when crack passed through whole sample, two parts of sample respectively rotate towards two sides, as shown in Figure 9a). However, after the peak load, the sample ‘FT-3’ still has a residual strength (about 58.8% of peak load) due to the bridging effect of steel fiber (Figure 6 and Figure 8c). During the loading process, the sample was in balance even after the crack has penetrated whole sample (As shown in Figure 9b, the sample can still sustain vertical load and big rotation does not happen).

**Figure 10:**
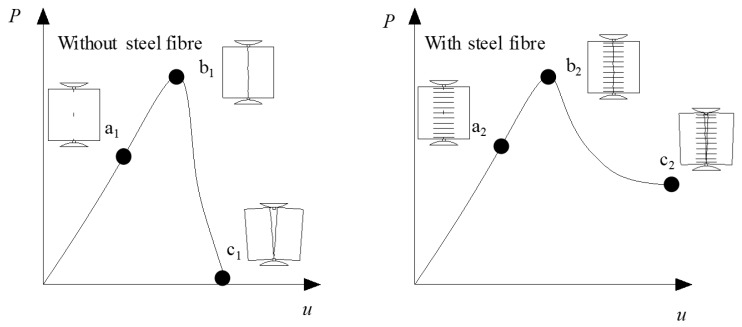
Schematic diagram for tensile-splitting tests on cubical samples without and with steel fiber, ‘a’, ‘b’ and ‘c’ indicates initial cracking state, peak load state and post-failure state, respectively.

Schematic diagram for tensile-splitting tests on cubical samples is shown in Figure 10. The testing process is divided into pre-cracking stage, pre-peak stage and post-failure stage. The load versus displacement and corresponding damage phenomenon at some key points are indicated by ‘a’, ‘b’ and ‘c’ in Figure 10. Subscript 1 and 2 represents cube without fiber and with fiber, respectively. The obvious difference is that the bridging effect of steel fiber enables the sample at stage ‘c_2_’ to still sustain load with the existence of a crack.

## 4. Numerical Simulation

### 4.1. Geometry Numerical Model

**Figure 11:**
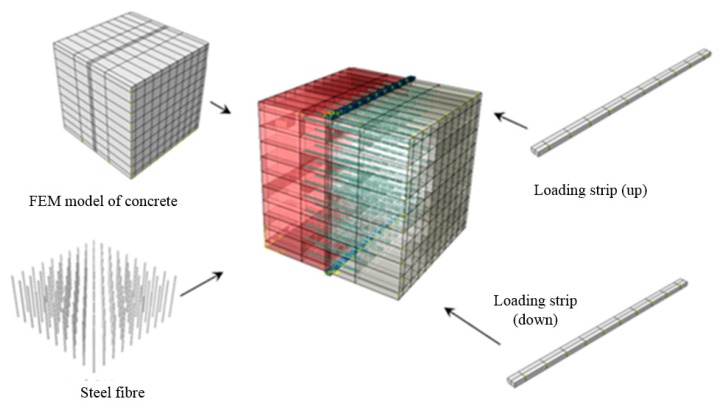
Finite element method (FEM) model of sample with steel fiber reinforced interface jointing two types of concrete.

**Figure 12:**
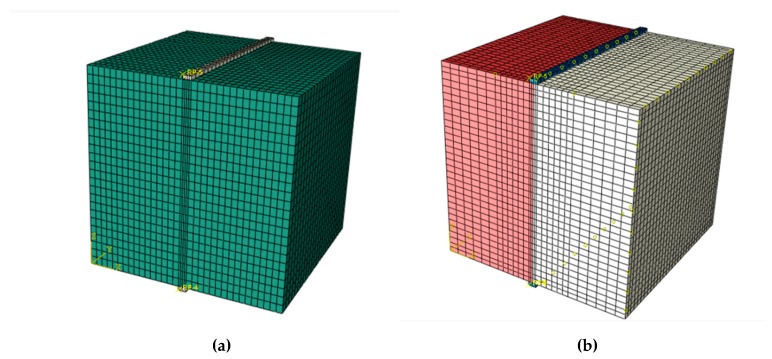
FEM mesh, (**a**) high strength concrete sample, low strength concrete sample and high strength/low strength mixed concrete sample; and (**b**) sample with steel fiber reinforced interface jointing two types of concrete.

The finite element software ABAQUS (2018) is employed to analyze tensile strength and failure process of the four types of samples (as shown in Figure 2). As shown in Figure 11, the finite element method (FEM) model of sample (FT) with steel fiber reinforced interface jointing two types of concrete includes loading strip, steel fiber and concrete. The geometries of every components are the same with the values used in lab tests. For the four FEM models, up and down loading strips adopt C3D8I element (8-node linear brick, incompatible modes), the concrete specimen adopts C3D8R element type(8-node linear brick, reduced integration with hourglass control), and the steel fiber adopts T3D2 element type (2-node linear 3-D truss). Steel fibers are embedded in concrete (“embedded” is a special constraint in ABAQUS which allows an object to be embedded into a “host” region of the model without the requirement of extra space. If a node of an embedded element lies within a host element, the translational degrees of freedom and pore pressure degree of freedom at the node are eliminated and the node becomes an “embedded node.” The translational degrees of freedom and pore pressure degree of freedom of the embedded node are constrained to the interpolated values of the corresponding degrees of freedom of the host element. Embedded elements are allowed to have rotational degrees of freedom, but these rotations are not constrained by the embedding [36]). The grid division of high strength concrete sample (HT), low strength concrete sample (LT) and high strength/low strength concrete sample with interface (OT) is the same (Figure 12a), including 20,928 elements and 23,625 nodes. The grid division of sample with steel fiber reinforced interface (FT) is shown in Figure 12b, with a total of 32,036 nodes and 28,756 elements.

### 4.2. Constitutive Model of Concrete

Concrete Damage Plasticity (CDP) model [36] in ABAQUS is a continuous plastic damage model for concrete, which employs isotropic elastic damage and isotropic tensile and compression plasticity theory to characterize the inelastic behavior of concrete. It can simulate the mechanical behavior of concrete under monotonic, cyclic or dynamic loads, and combine the plasticity associated with multiple sclerosis and isotropic elastic damage theory to describe the irreversible damage behavior in the process of fracture. Therefore, CDP model is adopted in the following simulations.

**Figure 13:**
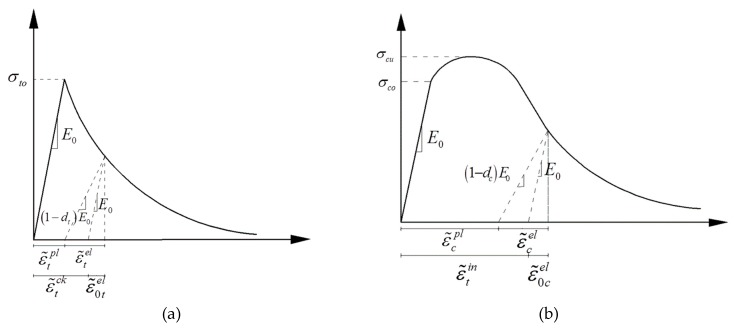
(**a**) Uniaxial tensile stress-strain relationship and the cracking strain ${\tilde{\varepsilon }}_{t}^{ck}$, (**b**) stress-strain relationship under uniaxial compression and compression inelastic strain diagram ${\tilde{\varepsilon }}_{c}^{in}$ [36].

The elastic parameters of concrete and steel fiber are demonstrated in Table 2. The CDP model assumes that concrete material is destroyed mainly by tensile cracking and compression crushing. The evolution of yield or failure surface is controlled by the two hardening variables ${\tilde{\varepsilon }}_{t}^{pl}$ and ${\tilde{\varepsilon }}_{c}^{pl}$, which represent the equivalent plastic strain of tension and compression respectively. The stiffness degradation of concrete materials due to damage is mainly manifested in different tensile and compressive yield strength, softening after tensile yield, hardening and softening after compression yield. Therefore, different damage factors are adopted to describe the stiffness degradation by CDP model, as shown in Figure 13.

For tensile behavior, concrete is assumed to be linearly elastic when the tensile stress is smaller than $\sigma_{t0}$ (Figure 13a). At this elastic stage, damage is not considered. When the failure stress is reached, cracks are generated. For compression behavior, concrete model is linear elasticity until the initial yield $\sigma_{c0}$ (Figure 13b) is achieved, followed by a hardening stage, and finally entering into strain softening stage after the ultimate stress $\sigma_{\mathrm{cu}}$. The stress-strain relationship of concrete under tension and compression is described by the following formulas [36]

(3) $$\sigma_{t}=(1-d_{t})E_{0}\left(\varepsilon_{t}-{\tilde{\varepsilon }}_{t}^{pl}\right),$$

(4) $$\sigma_{c}=(1-d_{c})E_{0}\left(\varepsilon_{c}-{\tilde{\varepsilon }}_{c}^{pl}\right),$$

where $d_{t}$ and $d_{c}$ is the damage factor for tensile and compressive condition, respectively. Implication of other parameters are shown in Figure 13.

The damage factors are calculated by the following formulas.

(5) $$d_{c}=1-\frac{\sigma_{c}E_{0}^{-1}}{\sigma_{c}E_{0}^{-1}+{\tilde{\varepsilon }}_{c}^{in}\left(1-1/b_{c}\right)},$$

(6) $$d_{t}=1-\frac{\sigma_{t}E_{0}^{-1}}{\sigma_{t}E_{0}^{-1}+{\tilde{\varepsilon }}_{t}^{ck}\left(1-1/b_{t}\right)},$$

(7) $$b_{t}={\tilde{\varepsilon }}_{t}^{pl}/{\tilde{\varepsilon }}_{t}^{ck},$$

(8) $$b_{c}={\tilde{\varepsilon }}_{c}^{pl}/{\tilde{\varepsilon }}_{c}^{in}.$$

In the calculations of concrete damage factors, the elastic modulus $E_{0}$ is the secant modulus, and according to the test analysis results of Birtel and Mark [37], $b_{t}$ = 0.1 and $b_{c}$ = 0.7. In addition, concrete is considered to have no damage and no plastic deformation before the stress reaching $\sigma_{t0}$ for tensile condition and $\sigma_{c0}$ for compressive condition. The simplified stress-strain relationships are listed as follows.

(9) $$\sigma_{t}=\{\begin{array}{cc} E_{0}\varepsilon & \varepsilon \leq \varepsilon_{t0}\\ \frac{\rho_{t}E_{c}\varepsilon }{\alpha_{t}{\left(\varepsilon /\varepsilon_{t0}-1\right)}^{1.7}+\varepsilon /\varepsilon_{t0}} & \varepsilon >\varepsilon_{t0} \end{array},$$

(10) $$\sigma_{c}=\{\begin{array}{cc} E_{0}\varepsilon & \varepsilon \leq \varepsilon_{c0}\\ \frac{\rho_{c}nE_{c}\varepsilon }{n-1+{\left(\varepsilon /\varepsilon_{cu}\right)}^{n}} & \varepsilon_{0}<\varepsilon \leq \varepsilon_{cu}\\ \frac{\rho_{c}E_{c}\varepsilon }{\alpha_{c}{\left(\varepsilon /\varepsilon_{cu}-1\right)}^{2}+\varepsilon /\varepsilon_{cu}} & \varepsilon >\varepsilon_{cu} \end{array},$$

(11) $$\rho_{t}=\frac{f_{t,r}}{E_{c}\varepsilon_{t,r}},$$

(12) $$\rho_{c}=\frac{f_{c,r}}{E_{c}\varepsilon_{c,r}},$$

(13) $$n=\frac{E_{c}\varepsilon_{c,r}}{E_{c}\varepsilon_{c,r}-f_{c,r}},$$

where $E_{c}$ is elastic modulus of concrete, $f_{t,r}$ represents uniaxial tensile strength of concrete, $\alpha_{t}$ is the parameter value of the descending section of the uniaxial tensile stress-strain curve of concrete, $f_{c,r}$ represents uniaxial compressive strength of concrete, $\alpha_{c}$ is the parameter value of the descending section of the uniaxial compression stress—strain curve of concrete.

In addition to above constitutive equations to describe the behavior of concrete, there are some other parameters should be used in the CDP model [36], as shown in Table 6.

### 4.3. Simulation Results

**Figure 14:**
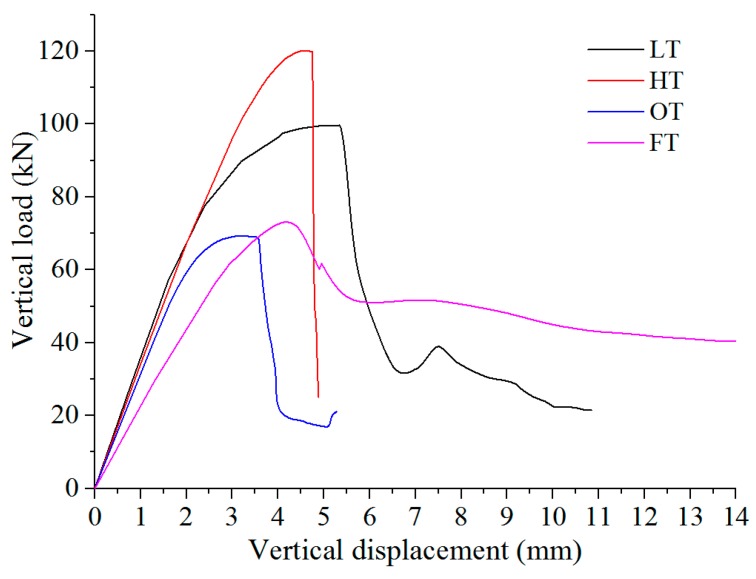
Numerical simulation results of vertical load-vertical displacement curves of samples during loading process.

The middle position of the upper loading strip is selected as the displacement monitoring point. The vertical load-vertical displacement curves of samples during the gradual loading process are obtained, as shown in Figure 14. The numerical simulation results show that the peak vertical loads of samples have the tendency of ‘HT’ > ‘LT’ > ‘FT’ > ‘OT’. The simulation value of peak load of ‘HT’, ‘LT’, ‘FT’ and ‘OT’ is 120.0 kN, 99.6 kN, 73.1 kN and 69.3 kN, respectively. In addition, the samples of ‘HT’, ‘LT’ and ‘OT’ exhibit typical brittle failure with a sudden drop in load after peak point. However, the sample ‘FT’ has a certain amount of residual strength due to the reinforcement of steel fiber. It can be concluded that the simulation results are in good agreement with the experimental results.

**Figure 15:**
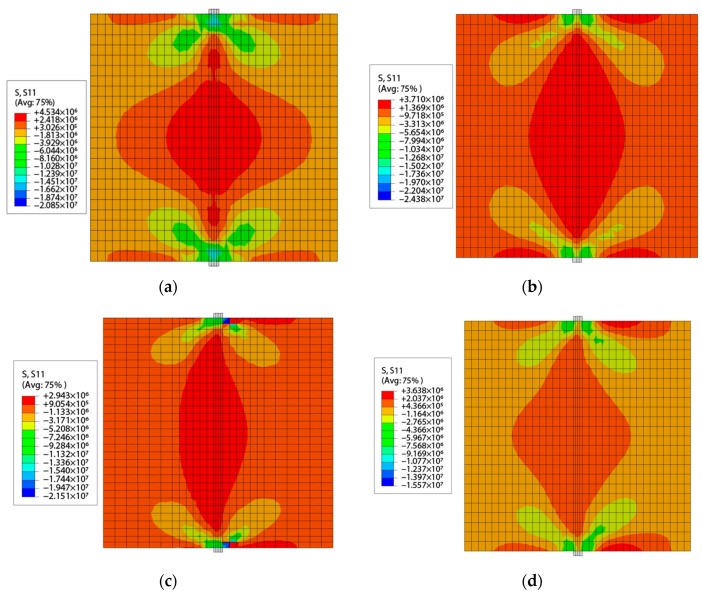
The cloud diagram of horizontal stress at the peak vertical load, (**a**) ‘HT’ sample, (**b**) ‘LT’ sample, (**c**) ‘FT’ sample, (**d**) ‘OT’ sample.

The contour of horizontal stress during the peak vertical load are shown in Figure 15. The tensile stress is generated inside samples between up and down loading strips, in the middle of the sample, the cracks first occurs due to the existence of tensile stress concentration, and then it develops to both ends, eventually leads to splitting failure of samples (the CDP model reflects the development of cracks in ABAQUS by tensile damage and compression damage changes). The magnitude of horizontal stress (tensile stress) has the similar tendency of ‘HT’ > ‘LT’ > ‘FT’ > ‘OT’. For samples of ‘FT’ and ‘OT’, the horizontal stress is not symmetrically distributed in the model (Figure 15c,d) although the geometry shape and the load are symmetrical, which can be attributed to existing of interface and being made of two kinds of concrete.

## 5. Conclusions

A series of tensile splitting tests and numerical simulations are performed on the high strength concrete, the low strength concrete, and cementation of high and low concretes (with and without steel fiber reinforced interface). The tensile splitting strength, initiation cracking load and damage characteristics are analyzed. According to the tests, the following conclusions can be drawn:

- The tensile splitting strength and initial cracking load for four types of cubical samples has the law of ‘HT’ > ‘LT’ > ‘FT’ > ‘OT’ (high strength concrete cube > low strength concrete cube > cube with interface reinforced by steel fiber > cube with interface without steel fiber). The average tensile splitting strength of ‘FT’ has increased about 10.9% compared with that of ‘OT’ due to the reinforced effects of steel fiber (the area percentage of fiber on the interface is about 0.35%). The ratio of average tensile splitting strength of ‘OT’ (interface) versus ‘HT’ and ‘OT’ versus ‘LT’ is about 48.8% and 67.4%, respectively. Therefore, the strength reduction due to the existence of the interface must be considered for designing and predicting performance of the structure.
- The tensile splitting strength is closely related to roughness of cracking surface and fractured aggregates (Higher tensile splitting strength exists if roughness is bigger and more aggregates are fractured).
- The bridging effect of steel fiber can reinforce the interface and results in a bigger peak load and residual strength. Especially, in contrast to other samples without steel fiber, the interface reinforced by steel fiber can still sustain load with existing of cracks in post-failure stage.
- The numerical method can not only correctly exhibit the macroscopical mechanical behavior of samples, but also showing corresponding microscopic stress state. The stress state could not be symmetrically distributed when geometry and the load is symmetrical for samples, due to the existence of the interface composited by two types of material.

## Figures and Tables

**Table 1:** Mix proportions of concretes.

Mixtures	High Strength Concrete	Low Strength Concrete
Cement (kg/m^3^)	388	227
Water (kg/m^3^)	145	149
Fine sand (kg/m^3^)	623	795
Coarse aggregate (kg/m^3^)	1081	1098
Limestone powder (kg/m^3^)	108	68
Slag (kg/m^3^)	50	44
Admixture (kg/m^3^)	13.65	6.4

**Table 2:** Basic mechanical parameters.

**Concrete Samples**	**Compressive Strength (MPa)**	**Elastic Modulus (GPa)**	**Poisson’s Ratio**	**Unit Weight (kg/m^3^)**
HC-1	69.1	45.2	0.14	2406
HC-2	65.4	44.7	0.15	2397
HC-3	70.4	47.5	0.14	2411
Average	68.3	45.8	0.14	2405
LC-1	36.2	39.5	0.14	2390
LC-2	35.3	38.4	0.15	2381
LC-3	31.6	37.9	0.16	2370
Average	34.4	38.6	0.15	2380
**Materials**	**Length (cm)**	**Diameter (mm)**	**Tensile Strength (MPa)**	**Elastic Modulus (GPa)**
Steel fiber	6	1	600	200

**Table 3:** Tensile splitting test results for four types of samples.

Samples	$P_{\max}$ (kN)	$\sigma_{ct}$ (MPa)	Average $\sigma_{ct}$ (MPa)
HT-1	137.313	3.885	3.697
HT-3	123.300	3.489	
HT-5	131.344	3.716	
LT-1	93.622	2.649	2.679
LT-3	93.461	2.644	
LT-4	96.940	2.743	
OT-1	64.376	1.821	1.805
OT-4	65.572	1.855	
OT-5	61.423	1.738	
FT-1	72.428	2.049	2.002
FT-2	71.641	2.027	
FT-3	70.373	1.991	
FT-4	68.620	1.942	

**Table 4:** The value of initial cracking points for different samples.

Samples	Initial Cracking Point $P_{i}$ (kN)	Peak Loads $P_{\max}$ (kN)	$P_{i}$/$P_{\max}$
HT-5	106.283	131.344	80.9%
LT-1	58.806	93.622	62.8%
FT-3	35.754	70.373	50.8%
OT-4	32.114	65.572	49.0%

**Table 5:** The different percentages of fractured areas.

Sample	$A_{a}/A_{total}$ (%)	$A_{b}/A_{total}$ (%)	$A_{c}/A_{total}$ (%)
HT-5	30.9	3.9	65.2
LT-1	12.0	15.5	72.5
OT-4	3.7	5.3	91.0

**Table 6:** Other parameters of the Concrete Damage Plasticity (CDP) model.

ψ	ϵ	$\sigma_{b0}/\sigma_{c0}$	$K_{c}$	μ
30°	0.1	1.16	2/3	0.0005

Where ψ is expansion angle, ϵ is flow potential offset, $\sigma_{b0}/\sigma_{c0}$ is ratio of ultimate compressive strength of two axes to ultimate compressive strength of one axle, $K_{c}$ is the ratio of the second stress invariant on the tension meridian plane to the compression meridian plane, and μ is viscosity coefficient.
